# Medical News

**Published:** 1855-12

**Authors:** 


### THE MEDICAL EXAMINER.

PHILADELPHIA, DECEMBER, 1855.

### MEDICAL NEWS.

A Consumption Hospital, the first of the kind in the United States, has been recently organized in New York, by the election of Dr. J. H. Griscom, as President, and Dr. A. H. Jones, as Secretary. Buildings will be erected as soon as the necessary funds are raised.

We regret to notice the death of Professor Johnston, of Durham, a well known Chemist and writer on scientific subjects.

Professor Allman, of Dublin, has been elected to the Chair of Natural History, in the Univerity of Edinburgh, vacated by the death of Prof. Forbes. Dr. Laycock, of York, a well known and distinguished writer, has succeeded, after a vigorous canvass, in obtaining the Chair of the Practice of Physic in the same Institution. His strongest competitors were Professor Bennett and Dr. Wood, of Edinburgh. Professor Alison, the previous incumbent, whom ill-health caused to retire, has been appointed Emeritus Professor.

British Mortality in England and the Crimea.—If all the deaths of British soldiers in the Crimea during the last three months were added to the deaths in England, the sum would be less by some twenty thousand than the deaths registered in England during the three summer months of 1854. In fact, it results from the Report of the Registrar General, for the Quarter ending September, 1855, that the summer months of the present year have been unusually favorable as regards health, notwithstanding the undoubted disadvantage under which the poor have suffered in respect to the price of provisions. The causes assigned for the improvement in the public health, by the District Registrars, are various, but they are quite in unison in respect to the inestimable advantages accruing from sanitary improvements. The deaths in the summer quarters of the five years 1851–55 were as follows:—

| 1851. | 1852. | 1853. | 1854. | 1855. |
|---|---|---|---|---|
| 91,499 | 100,382 | 92,201 | 113,939 | 87,934 |

It is a curious circumstance, in reference to the means of livelihood of the people, that while in the interval of two years the price of wheat has risen 48 per cent., and that of beef 15 per cent., there has been a fall of 37 per cent. in the price of potatoes, an article of large consumption among all classes, and which has operated as a set-off against the high price of other articles; thus, perhaps, partly accounting for the fact that the public health is better than in the summer of 1853. In reference to the Meteorology of the quarter, Mr. Glaisher reports that the departures from the average values of the temperature have in no cases been large during the quarter, and that other subjects of investigation have been about their average. Such being the facts of the case, it remains only that, eschewing the insignificant few who, for peculiar reasons, are *sans* olfactories, we steadily pursue the work of sanitary reform, having confidence in the aphorism, "*Aide-toi, et le Ciel t'aidera.*" —*London Medical Times.*

---

CARD OF THE COMMITTEE ON PRIZE ESSAYS OF THE AMERICAN MEDICAL ASSOCIATION.—At a meeting of the American Medical Association, held in Philadelphia, May, 1855, the undersigned were appointed a Committee to receive voluntary communications on Medical subjects, and to award prizes in accordance with the regulations of that body.

Each communication intended to compete for a prize, must be addressed to the Chairman of the Committee, at Ann Arbor, Michigan, before March 20th, 1856, and must be accompanied by a sealed packet, containing the name of the author, and marked exteriorly by a sentence or motto, corresponding with one upon the essay; which packet will not be opened unless the essay belonging to it is successful in obtaining a prize. Unsuccessful papers will be returned on application, after the adjournment of the Meeting of the Association, in Detroit, in May next.

A. B. PALMER, M. D. (Chairman), SAMUEL DENTON, M. D.,
A. R. TERRY, M. D., A. SAGER, M. D., S. H. DOUGLASS, M. D.,
C. L. FORD, M. D., E. ANDREWS, M.D.

---

We have been desired to copy the following notice. We sincerely hope that its object, which all must allow to be a very laudable one, will be crowned with success.

PHILADELPHIA, NOVEMBER, 1855.

DEAR DOCTOR:—At the last meeting of the Pennsylvania State Medical Society the following Resolution was adopted:—

"*Resolved*, That the Corresponding Secretary be, and he is hereby instructed to address a circular to the prominent Physicians in those portions of the State in which no County Medical Societies exist, urging upon them the importance of organizing such Societies without delay."

In this age, the influence of associated action is invoked by every class of the community which has interests to protect, or objects to accomplish; it cannot, therefore, be regarded as singular, that Physicians should seek its aid to protect the interests of their Profession, which, in this State, the law leaves entirely unguarded.

We do not ask your assistance to obtain Legislative Protection, but we earnestly urge you to assist us in protecting ourselves: organize a County Medical Society without delay, enroll the names of the educated, moral, and regular Physicians, and exclude from the brotherhood the ignorant, the irregular, and the unprincipled; then will your brethren at a distance know to whom to apply for advice and assistance, and will co-operate with you in all measures calculated to extend the usefulness of our high calling, and to elevate, dignify and adorn it. Bring to your County Society the results of your observations and experience in Medicine and its kindred sciences; collate them and send them to the State Society for common improvement, and receive in return the observations and experience of Physicians in other parts of the State; thus will all be benefitted, and much valuable information be preserved and circulated.

The scandal of our profession is, and always has been, that Physicians not only differ in their treatment of disease, but that they do not live together in social harmony. The formation of a County Society leads to more frequent professional and social intercourse, and thus elevates the character of the profession by discouraging the spirit of ungenerous rivalry, reconciling enemies, and healing those differences which arise from misrepresentation or fancied injury.

Our annual reunions are, moreover, seasons of pleasure as well as profit, for in them, warm-hearted, accomplished, and distinguished Physicians, from all parts of the State, meet in social as well as scientific fellowship.

The next meeting of the State Society will be held in Philadelphia, on the last Wednesday in May next, when we will be happy to receive you as a delegate or individual member from your County, and will endeavor to make your sojourn in the Medical metropolis of the country as pleasant as possible.

Yours, very respectfully, THOS. H. YARDLEY,
*Corresponding Secretary of State Medical Society.*

NOTE.—The objects of the Pennsylvania State Medical Society, as declared by the Constitution, are "the elevation of professional character; the protection of the interests of its members; the extension of the bounds of medical science; and the promotion of all measures adapted to the relief of suffering, and to improve the health and protect the lives of the community."

The members consist of ASSOCIATES and DELEGATES; every member of a properly organized County Society is an associate; the delegates are elected by the County Societies in the proportion of one to every five of its members; but every County Society, however small, is entitled to one delegate.

The Delegates meet annually, to transact the business of the Society. The first meeting was held in the City of Reading, in 1849, at which only ten County Societies were represented; now twenty-five Counties have formed Societies, and we hope at the next meeting to have delegates from every County in the State.

A volume of the *Transactions* is published annually, containing the names of the Officers and Members of the County Societies, their reports, and much other valuable information. A few volumes of these *Transactions* remain on hand, and will be forwarded to the address of any Physician who is willing to take the first step for the formation of a County Society; or any other information on the subject will be given on application to

THOS. H. YARDLEY,
*No.* 381 *Arch Street, Philadelphia.*

